# Structural changes in the upper trapezius muscle of fibromyalgia patients identified by quantitative ultrasonography: a cross-sectional study

**DOI:** 10.1007/s00296-025-05871-x

**Published:** 2025-04-22

**Authors:** Hatice Betigul Meral, Aylin Rezvani, Sena Tolu, Ahmet Usen, Muhammed Furkan Dasdelen

**Affiliations:** 1 Faculty of Medicine, Department of Physical Medicine and Rehabilitation, Istanbul Medipol University, Istanbul, Türkiye; 2 Faculty of Medicine, Istanbul Medipol University, International School of Medicine, Istanbul, Türkiye

**Keywords:** Fibromyalgia, Hyperalgesia, Ultrasonography, Diagnostic imaging, Pain

## Abstract

The heterogeneity of symptoms among patients with fibromyalgia (FM) makes the development of standardized diagnostic criteria challenging. No imaging technique has reliably shown FM-related muscle changes to aid clinical assessment. This study aimed to quantitatively analyze the upper trapezius muscle in FM patients using B-mode ultrasonography and blob analysis and to examine its correlation with clinical parameters. A total of 34 female FM patients and 34 healthy controls were included in this cross-sectional study. B-mode ultrasonography was used to image the dominant-side upper trapezius muscle, and MATLAB-based blob analysis was performed to assess blob size, blob count, and echointensity. These measurements were correlated with disease severity indices, including the Central Sensitization Inventory (CSI), Visual Analog Scale (VAS) for pain, Fibromyalgia Impact Questionnaire (FIQ), 36-Item Short Form Survey (SF-36), and Beck Depression and Anxiety Inventories (BDI, BAI). FM patients had significantly higher total blob size (*p* < 0.001) and blob size per mm² (*p* < 0.001) than controls. Echointensity was significantly increased in the FM group (*p* = 0.009). Total blob size showed a moderate positive correlation with CSI scores (*p* = 0.002). Regression analysis indicated that pain-VAS was a significant predictor of total blob size per mm² (*p* < 0.001). Blob analysis demonstrated quantifiable muscle alterations in FM, supporting its potential role as an objective assessment tool. Given the correlation between muscle echotexture and FM severity, quantitative ultrasonography may contribute to a better understanding of FM pathophysiology.

## Introduction

Fibromyalgia (FM) is a chronic pain syndrome that affects a significant portion of the population worldwide, yet its pathophysiology remains elusive [1]. It is characterized by widespread musculoskeletal pain, fatigue, cognitive dysfunction, and sleep disturbances, significantly impacting patients’ quality of life [2, 3]. The global prevalence of FM is estimated to be 2–3%, with a higher prevalence in women [4, 5]. Despite its substantial burden, the exact mechanisms underlying FM are not yet fully understood, though both genetic and environmental factors are believed to play a role [6, 7].

In recent years, the role of central pain mechanisms and central sensitization in FM has gained increasing attention [8, 9]. As a key contributor to chronic pain pathophysiology, central sensitization syndrome is now recognized as a fundamental feature of FM [9].

The 1990 American College of Rheumatology (ACR) diagnostic criteria for FM are based on the assessment of tender points, prompting numerous studies that investigate the specific muscles involved in these regions [10–12]. Some studies indicate that these muscles exhibit elevated concentrations of anaerobic glycolysis end products, which may contribute to oxidative metabolic damage resulting from localized hypoxia [13, 14]. A biopsy study of tender points in the upper trapezius of FM patients revealed decreased levels of adenosine triphosphate (ATP), adenosine diphosphate (ADP), and phosphocreatine, alongside increased concentrations of adenosine monophosphate (AMP) and creatine [15, 16]. Furthermore, studies on exercise in patients with FM have reported reduced muscle blood flow compared to healthy controls [17, 18]. These findings suggest structural and functional changes in muscle tissues identified as tender points in patients with FM.

Ultrasonography is a reliable, noninvasive imaging modality that provides high-resolution evaluation of superficial musculoskeletal structures and enables dynamic assessments [19]. Traditional approaches to muscle tissue analysis have predominantly relied on visual inspection or assessments based on muscle thickness measurements. However, quantitative ultrasonographic techniques have gained increasing attention as they allow for a more detailed and objective evaluation of muscle architecture [20, 21]. Among these methods, blob analysis is a technique specifically designed to quantify structural patterns in ultrasonographic images by assessing spatial connectivity and echointensity homogeneity [22, 23]. Blobs are defined as spatially contiguous regions with similar echogenic properties, and their size, count, and distribution serve as quantitative parameters for characterizing muscle microstructure in ultrasonographic imaging [22].

Nielsen et al. used B-mode ultrasonography to evaluate healthy shoulder and thigh muscles using blob analysis, demonstrating its potential to quantitatively distinguish muscle groups [22]. Another study used blob analysis to identify tissue changes in the upper trapezius of patients with myofascial pain syndrome [24]. However, despite studies utilizing these novel imaging techniques in the mentioned and various other conditions, no studies to date have employed blob analysis to investigate structural muscle changes in FM.

Based on these findings, this study aimed to quantitatively analyze the upper trapezius muscle using B-mode ultrasonography in patients with FM, compare blob analysis results (blob size, blob count, and echointensity) between patients with FM and healthy controls, and evaluate their relationships with FM parameters, particularly the severity of central sensitization.

## Methods

This cross-sectional study was conducted between February 2021 and November 2021 in our institution. It included 34 patients diagnosed with FM based on the 2016 revised fibromyalgia classification criteria of the ACR and 34 healthy controls who met the inclusion criteria. This study was approved by the appropriate Ethics Committee (approval number E-10840098-772.02-6754) and adhered to the STROBE guidelines.Informed consent was obtained from all participants.

The inclusion criteria were women, aged between 18 and 60 years, having been followed up for at least 6 months with an FM diagnosis, and having the ability to read and sign an informed consent form. The exclusion criteria were the presence of diagnosed endocrine, neuromuscular, infectious, or rheumatologic diseases; cervical radiculopathy; history of malignancy; neck surgery; trauma; pregnancy or lactation; or male sex.

All the patients were asked about their medications, demographic data, symptom duration, dominant extremities, duration of complaints, duration of diagnosis, and comorbidities.In patients with FM, pain severity was assessed using the Widespread Pain Index (WPI) included in the 2016 revised ACR criteria, whereas symptom severity was evaluated using the Symptom Severity Scale (SSS) from the same criteria. Pain, fatigue, and sleep were assessed using a Visual Analog Scale (VAS). Quality of life was evaluated using the 36-Item Short Form Survey (SF-36), physical activity levels were measured using the International Physical Activity Questionnaire-Short Form (IPAQ-SF), and physical function using the Fibromyalgia Impact Questionnaire (FIQ). Depression and anxiety levels were measured using the Beck Depression Inventory (BDI) and the Beck Anxiety Inventory (BAI), respectively. The degree of central sensitization was evaluated using the Central Sensitization Inventory (CSI) Part A, and the presence of other central sensitization syndromes was assessed using the CSI Part B.

### B-Mode ultrasonographic evaluation of the upper trapezius muscle

Ultrasound images of the upper trapezius muscle were obtained in a blinded manner by a physician with five years of experience in musculoskeletal ultrasonography. A Philips Lumify ultrasound device with an L12-4 linear transducer (Philips Medical Systems, Bothell, WA, USA) was used for imaging.

The gain, dynamic range, and depth settings of the ultrasound device were standardized for both the patient and healthy groups. The depth was set to 5 cm and the time gain compensation was adjusted to 60 based on previous studies [14].

**Fig. 1:**
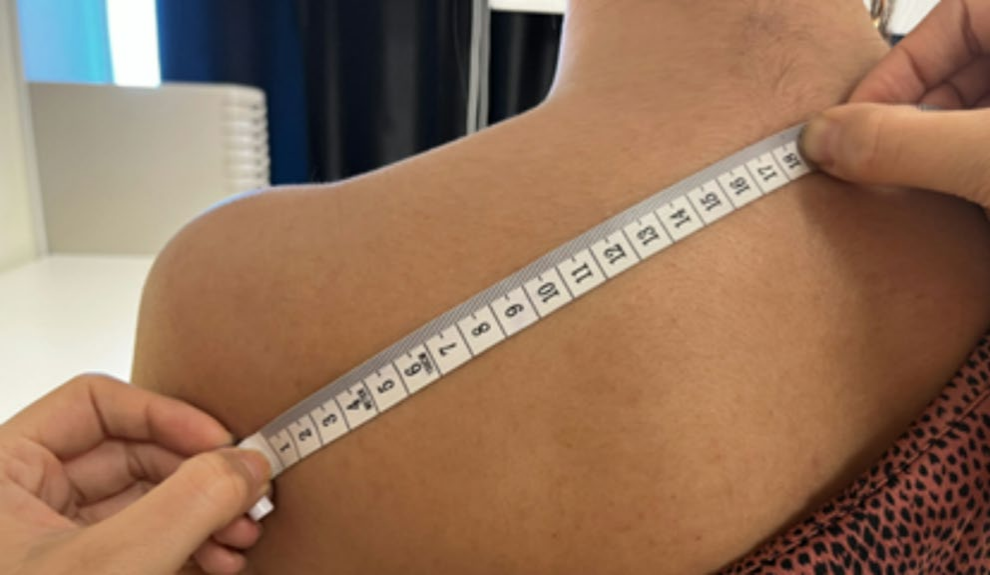
Measurement of the distance between the C7 spinous process and the acromioclavicular joint to position the transducer

To obtain images of the upper trapezius muscle, the participants were asked to sit on a chair with their forearms resting on their thighs and cervical spine in a neutral position. The transducer was placed on the side of the dominant extremity, at the midpoint between the C7 spinous process and the acromioclavicular joint, as described in previous studies (Fig. 1). The transducer was aligned longitudinally along the muscle fibers to capture the longest axis of the trapezius muscle. Ample gel was applied between the transducer and skin to prevent muscle deformation or changes in echointensity or cross-sectional thickness.

The transducer angle relative to the skin was adjusted to obtain the highest echointensity image, and three consecutive static B-mode ultrasound images were captured without moving the transducer.

### Quantitative ultrasonography image analysis protocol

For each image, the muscle area within the region of interest (ROI) was manually delineated to exclude the fascia and other tissues. The resolution of the ultrasound images ranged from 580 × 125 to 540 × 110 pixels. The images were transferred to MATLAB and Statistics Toolbox Release (MATLAB 2016b; MathWorks, Inc., Natick, MA, USA). A blob texture analysis algorithm was applied based on the methodology developed by Nielsen et al. [14]. The pixels in each image were assigned grayscale values ranging from 0 (darkest) to 255 (brightest) and the echointensity of each image was calculated. Areas with echointensity values within the 95th and 99th percentiles derived from images of healthy participants were identified as the regions of interest.

**Fig. 2:**
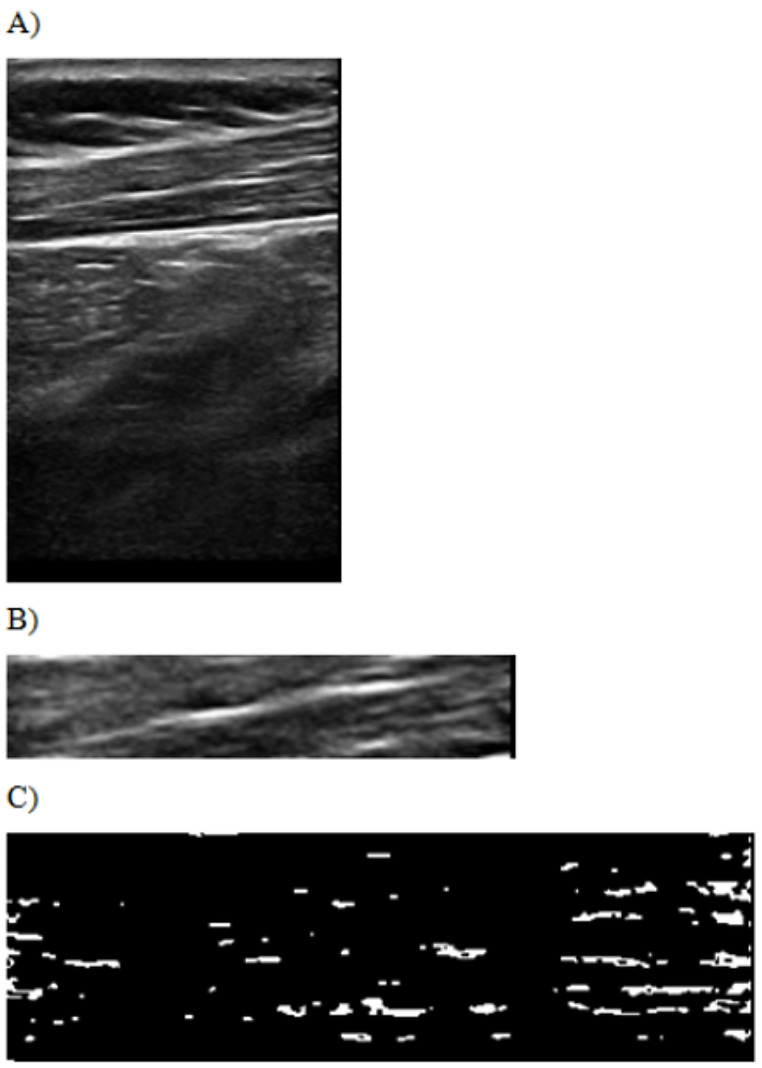
B-mode ultrasound image of the upper trapezius muscle (**A**). The region of interest (ROI) area of the upper trapezius muscle (**B**). Blob positions within the ROI after thresholding (**C**)

Initially, the ROI images were thresholded using MATLAB 2016b software. Thresholding involves converting the grayscale of the original image into a binary scale. Grayscale values below the predetermined threshold were assigned a value of 0, whereas values above the threshold were assigned a value of 1. This process created a binary “image” system where areas with a value of 1 represented blobs, defined as spatially connected regions of pixels with similar echointensity values. Blobs were identified in regions within the 95th and 99th percentiles of echointensity values that exceeded the threshold (Fig. 2).

### Statistical analysis

Descriptive statistics were used to summarize continuous (mean, standard deviation, minimum, median, and maximum) and categorical variables (frequencies and percentages). Pearson correlation analysis was performed to compare normally distributed continuous variables, Spearman’s rho was used for non-normally distributed variables, independent Student’s t-tests were used to compare two normally distributed variables, the Mann–Whitney U test was applied for non-normally distributed variables, chi-square or Fisher’s exact tests were used to assess relationships between categorical variables, and multiple linear regression analysis was applied to examine the effects of independent variables on continuous dependent variables. To reduce the risk of Type I errors due to multiple comparisons, the Benjamini-Hochberg False Discovery Rate (FDR) correction was applied to the p-values reported in the correlation and regression analyses.

A statistical significance threshold of *p* < 0.05 was used for all analyses. All statistical analyses were conducted using the MedCalc Statistical Software version 12.7.7 (MedCalc Software bvba, Ostend, Belgium; http://www.medcalc.org; 2013).

## Results

A total of 34 FM patients and 34 healthy controls who met the inclusion criteria were included in the study. The median age of the FM group was 45 years, whereas that of the healthy group was 39 years. The median body mass index (BMI) in the FM group was 28.24, compared with 25.82 in the healthy group. There were no statistically significant differences between the groups in terms of age, BMI, marital status, or educational level (*P* > 0.05; see Table 1).

**Table 1:** Comparison of relevant data between FM patients and healthy individuals

Characteristics	Healthy *N* = 34	Fibromyalgia patients *N* = 34	*p* value	FDR-adjusted *p*-value
SF-36 *				
Physical functioning	100 (70–100)	50 (1–95)	**< 0.001** ^**1**^	**0.002**
Physical role	100 (0–100)	0 (0–100)	**< 0.001** ^**1**^	**0.002**
Bodily pain	77.5 (22.5–100)	32.5 (0–100)	**< 0.001** ^**1**^	**0.002**
General health	70 (25–95)	25 (5–90)	**< 0.001** ^**1**^	**0.002**
Vitality	55 (25–85)	25 (0–100)	**< 0.001** ^**1**^	**0.002**
Social functioning	75 (0–100)	50 (0–100)	**0.011** ^**1**^	**0.015**
Emotional role	100 (0–100)	0 (0–100)	**< 0.001** ^**1**^	**0.002**
Mental health	64 (36–92)	42 (8–100)	**< 0.001** ^**1**^	**0.002**
BAI*	9 (0–23)	19 (3–61)	**< 0.001** ^**1**^	**0.002**
BDI**	7 ± 4	18 ± 9	**< 0.001** ^**2**^	**0.002**
IPAQ-SF Physical activity level, n(%)				
Physically Inactive	22 (64.7%)	9 (26.5%)	**0.004** ^**3**^	**0.007**
Low Physical Activity Level	11(32.4%)	23 (67.6%)		
Physical Activity Level Sufficient	1(2.9%)	2 (5.9%)		
IPAQ-SF Total physical activity*	2649.5 (0-25830)	3621 (0-52416)	**0.015** ^**1**^	**0.019**

Note: Values of *p* < 0.05 were accepted as significant and are marked in bold

Abbreviation: SF-36: 36-Item short form survey, BAI: Beck anxiety inventory, BDI: Beck depression inventory, IPAQ-S: International physical activity questionnaire short form, n: number of participants, FDR: False discovery rate

* Median (minimum-maximum), ** Mean ± SD, ^1^Mann-Whitney U test,^2^Student t test

^3^Fisher’s Exact test

Data from clinical evaluations, including disease-specific and other scale parameters, are presented in Tables 1 and 2. Significant differences were observed between the groups in terms of sleep-VAS, fatigue-VAS, pain-VAS, BAI score, BAI severity score, BDI score, and BDI severity score (*p* < 0.05).

**Table 2:** Disease and scale data of FM patients

Characteristics	Fibromyalgia patients *N* = 34
Complaint duration (Months)**	72 (1–360)
Diagnosis Duration (Months)**	24 (1–240)
Drugs, n (%)	
None	19 (55.9)
Duloxetine	8 (23.5)
Duloxetine + Pregabalin	3 (8.8)
Pregabalin	3 (8.8)
Amitriptyline	1 (2.9)
FM-WPI**	17 (6–19)
FM-SSS**	10 (2–13)
Sleep- VAS**	6 (1–9)
Fatique-VAS**	8 (4–9)
Pain-VAS**	8 (3–9)
FIQ**	66,97 (29,19–87,8)
FIQ Cut Off, n (%)	
< 50	9 (26.5%)
≥ 50	25 (73.5%)
CSI total score*	50 ± 16
CS severity level, n (%)	
Subclinic	3 (8,8)
Mild	7 (20,6)
Moderate	6 (17,6)
Severe	9 (26,5)
Extreme	9 (26,5)

Abbreviation: CS: Central sensitization, CSI: Central sensitization inventory, FIQ: Fibromyalgia impact questionnaire, SSS: Symptom severity, VAS: visual analogue scale; WPI: widespread pain index, n: number of participants; SD: Standart deviation

** Median (minimum-maximum), * Mean ± SD

Comparing the two groups, the distribution of BAI and BDI scores indicated a higher proportion of moderate-to-severe levels in patients with FM. According to the FIQ results, 25 patients with FM had scores above the cutoff, with 23 classified as severe, 10 as moderate, and 1 as mild.

Significant differences were also observed between the groups in the SF-36 subscales, including Physical Function, Physical Role, Bodily Pain, General Health, Vitality, Social Functioning, Emotional Role, and Mental Health parameters, as well as in the CSI total score and IPAQ-SF scores (*p* < 0.05). Patients with FM showed lower medians in SF-36 subscales, including Physical Function, Physical Role, pain, General Health, Vitality, Social Functioning, Emotional Role, and Mental Health, but higher IPAQ-SF scores than healthy controls.

The median duration of diagnosis for patients with FM was 24 months, whereas the median duration of complaints was 72 months. In the FM group, eight patients received duloxetine, three with pregabalin, three with duloxetine-pregabalin combination therapy, and one with amitriptyline (Table 2).

**Fig. 3:**
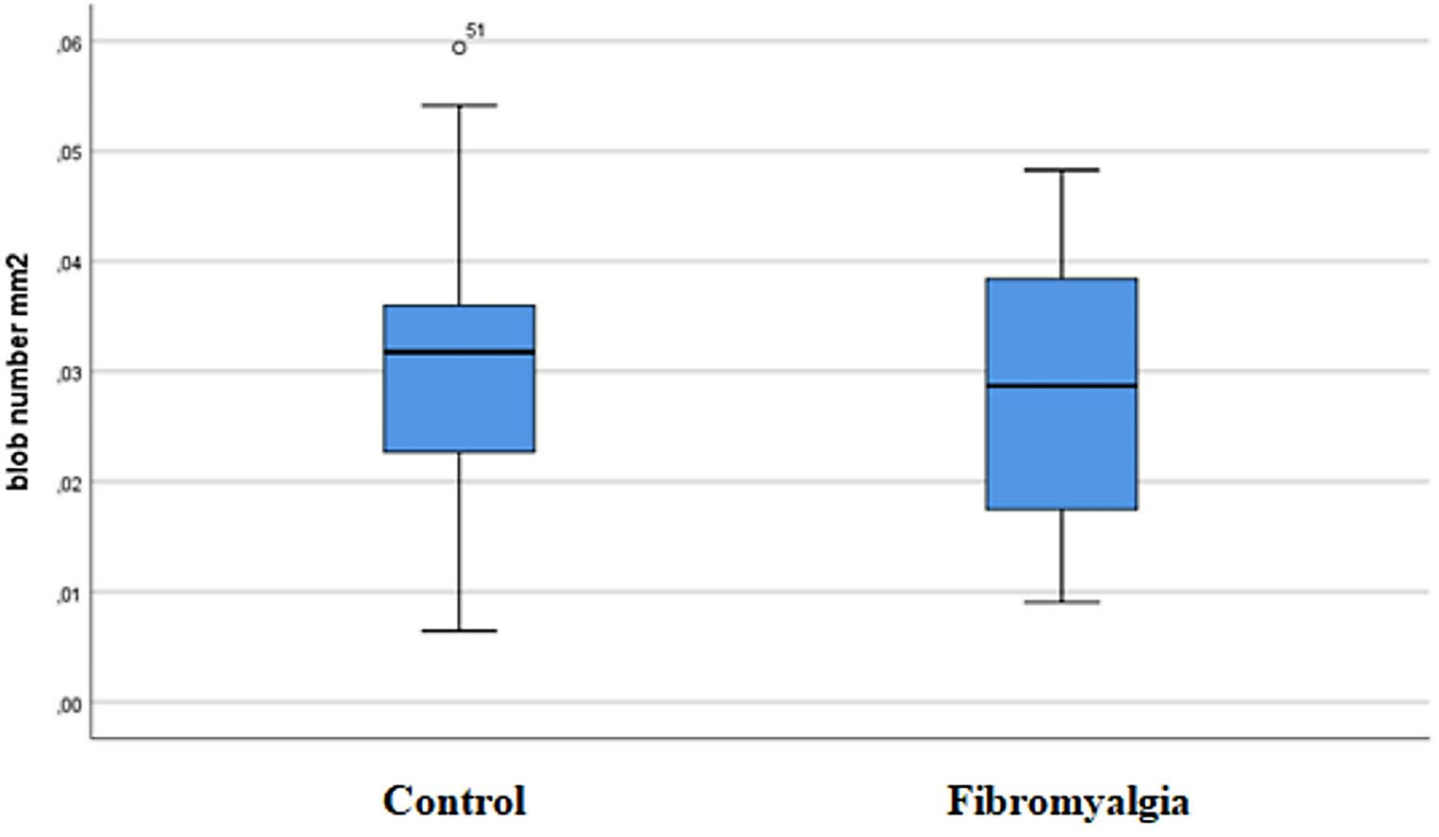
Distribution of blob count/mm² in healthy and fibromyalgia (FM) groups

The total blob size (2437.68 ± 750.61 mm²) and blob size/mm² (1811.3 ± 557.74 mm²) distributions were significantly higher in the FM group than in healthy controls (1680.21 ± 390.59 mm² and 1248.47 ± 290.23 mm², respectively; *p* < 0.001). Regarding the echointensity distribution, there were statistically significant differences between the FM and healthy groups (*p* = 0.009). The mean echointensity was higher in patients with FM (78.48 ± 11.42) than in healthy controls (71.95 ± 11.35). Quantitative ultrasonography results and group comparisons are presented in Table 3. When examining the distribution of blob counts/mm² between the FM and healthy groups, the FM group exhibited a much wider range of blob counts/mm² (49.47 ± 24.73) than the healthy controls (35.71 ± 13.16) (Fig. 3).

**Table 3:** Quantitative ultrasound parameters: differences between FM patients and healthy groups

Characteristics	Healthy *N* = 34	Fibromyalgia patients *N* = 34	*p* value	FDR-adjusted *p*-value
Blob Count*	37 (8–66)	42 (19–102)	0.067^1^	0.067
Total blob size*	1673 (870–2561)	2337 (1216–4077)	**< 0.001** ^**1**^	**0.002**
Total blob size/mm²*	1243.11 (646.45-1902.94)	1736.49 (903.54-3029.39)	**< 0.001** ^**1**^	**0.002**
Echointensity*	71.97 (46.88–98.45)	81.3 (49.45-100.51)	**0.009** ^**1**^	**0.013**

Note: Values of *p* < 0.05 were accepted as significant and are marked in bold

Abbreviation: FDR: False discovery rate

n: number of participants

* Median (minimum-maximum) ^1^Mann–Whitney U test

Correlation analysis revealed a weak but statistically significant positive correlation between blob count and BMI, fatigue-VAS, pain-VAS, and CSI total score (*p* < 0.05). The total blob size was positively correlated with weight and sleep-VAS at a weak level; duration of complaints, duration of diagnosis, FM-SSS, fatigue-VAS, pain-VAS, BDI score, and CSI total score at a moderate level (*p* < 0.05).

Total blob size/mm² showed weak positive correlations with weight and sleep-VAS and moderate positive correlations with duration of complaints, duration of diagnosis, FM-SSS, fatigue-VAS, pain-VAS, BDI score, and CSI total score (*p* < 0.05). A weak positive correlation was also found between the blob counts/mm² and BMI (*p* < 0.05).

Echointensity was weakly positively correlated with weight, duration of diagnosis, sleep-VAS, fatigue-VAS, and CSI total score, and moderately positively correlated with BMI, duration of complaints, FM-SSS, pain-VAS, BAI score, and BDI score (*p* < 0.05).

Regression analysis included variables found to be significant in the correlation analysis, along with the medications used by patients. For blob count, blob size, and echointensity values, there were no issues with multicollinearity (Variance Inflation Factor < 10), and there was no autocorrelation (Durbin–Watson value < 2). All models were statistically significant (*p* < 0.05) and the backward method was used for variable selection.

Regression analysis revealed that a one-unit increase in pain VAS was associated with a 1.374-unit increase in blob count. A one-unit increase in the duration of diagnosis was associated with a 3.255-unit decrease in total blob size per mm², while a one-unit increase in pain VAS was also associated with an 83.706-unit increase in total blob size per mm². Additionally, each one-unit increase in the Physical Role domain (SF-36) decreased the total blob size per mm² by 3.829 units, while a one-unit increase in the General Health domain (SF-36) increased it by 7.406 units (Table 4).

**Table 4:** Regression analysis results

Predictor variables	Nonstandardized β	SE	Standardized β	*p* value	FDR-adjusted *p*-value
Predictor of blob count					
Pain-VAS	1.374	0.66	0.252	**0.041**	**0.045**
Predictor of total blob size/mm²					
Diagnosis Duration (months)	-3.255	1.412	-0.273	**0.024**	**0.028**
Pain-VAS	83.706	22.302	0.611	**< 0.001**	**0.002**
Physical role (SF-36)	-3.829	1.638	-0.309	**0.023**	**0.028**
General health (SF-36)	7.406	2.626	0.368	**0.006**	**0.010**
Predictor of echointensity					
BMI	0.525	0.259	0.237	**0.047**	**0.049**
General health (SF-36)	-0.146	0.052	-0.329	**0.007**	**0.011**

Note: Values of *p* < 0.05 were accepted as significant and are marked in bold

Abbreviation: SE: Standart error. BMI: Body mass index. SF-36: 36-Item short form survey, FDR: False discovery rate

For echointensity, a one-unit increase in BMI was associated with a 0.525-unit increase, whereas a one-unit increase in General Health (SF-36) was associated with a 0.146-unit decrease.

## Discussion

To our knowledge, this study is the first to assess the potential use of blob analysis in FM, introducing a novel ultrasonographic method for more objective muscle assessment. Considering previous diagnostic approaches and studies in FM, this research offers a quantitative imaging perspective to further investigate muscle alterations.

Diagnosing FM remains challenging owing to the symptom overlap with conditions such as myofascial pain syndrome and the non-specific nature of its clinical presentation [25]. Although terms, such as the WPI, SSS, and generalized pain were introduced with the revised 2016 criteria, it should be acknowledged that this assessment is still primarily based on subjective patient-reported outcomes [26]. Chronic muscle pain, stiffness, and fatigue are hallmark features of FM, making muscle structure evaluation a compelling target for understanding its pathophysiology. Initially, the diagnosis of FM largely relied on the assessment of tender points through pressure evaluation. The evaluation of tender points in patients with FM using ultrasonography can provide clinicians with reliable data, aiding in both diagnosis and monitoring, especially in conditions where the assessment of muscle pain is prioritized.

Although no specific laboratory or imaging tests currently exist for FM diagnosis, various studies have investigated structural and functional muscle changes using different methodologies [27, 28]. Henriksson et al. performed one of the early systematic studies investigating muscle alterations in FM, examining structural changes in the muscle tissue of patients diagnosed with FM [29]. Biopsy samples obtained from the tender points of the trapezius muscle revealed a focal loss of nicotinamide adenine dinucleotide (NADH) diaphorase activity, presenting a characteristic moth-eaten appearance. Additionally, electron microscopy demonstrated mitochondrial abnormalities, Z-band streaming in myofibrils, and cytoplasmic bodies. Another study by Kalyan–Raman et al. confirmed the presence of moth-eaten fibers in type I fibers of the trapezius muscle. Electron microscopy revealed selective type II fiber atrophy and subsarcolemmal deposition of glycogen and mitochondria [30]. Although these muscle biopsy studies provide valuable insights into the structural and metabolic alterations associated with FM, their invasive nature and limited practicality hinder their routine use in clinical practice.

A literature review revealed that research on microcirculatory dysfunction in FM suggests a significant link between impaired blood flow and development of muscular pain. Lund et al.identified irregular tissue oxygen pressure distribution in patients with FM compared to healthy controls, indicating inadequate oxygen delivery to tender point regions, potentially due to localized ischemia, suggesting that compromised microcirculation may trigger pain at specific sites by contributing to intramuscular nociceptor sensitization and amplifying pain perception [31]. In a study conducted by Sanderson et al., which evaluated the histological response of skeletal muscle to ischemia using electron microscopy, several changes were observed in both electron and light microscopy [32]. Concomitant fibroplastic activity was observed throughout this process, along with the production of new collagen fibers. These findings suggest that ischemic changes may lead to disruptions in the fundamental muscle structure.

Muscle dysfunction and central sensitization are interconnected in FM. Peripheral sensitization driven by tissue alterations lowers nociceptor thresholds and amplifies afferent fiber sensitivity [33]. Although inflammation is typically absent, nociceptors and elevated levels of substance P have been detected in FM muscle tissue [34, 35]. These peripheral changes create persistent nociceptive input, feeding into central sensitization and sustaining pain perception [36, 37]. Consistent with this, one of the key findings of our study was the significant positive correlation between blob analysis results and CSI scores in patients with FM, supporting the hypothesis that nociceptive stimuli, along with structural changes in muscle tissue, may contribute to central sensitization.

Ultrasound is becoming an essential part of rheumatology practice and research, offering a feasible and effective imaging technique for real-time recognition of anatomical changes. It has emerged as a critical tool in the early diagnosis of rheumatic diseases such as rheumatoid arthritis (RA), gout, and Sjögren’s syndrome, leading to its integration into their diagnostic classification criteria [38, 39]. In RA, ultrasound facilitates the early detection of synovitis, identifying subclinical inflammation that may not be apparent during clinical examination, thereby allowing for timely diagnosis and treatment initiation [40].

Similarly, in spondyloarthritis (SpA), ultrasound can detect early structural changes such as enthesitis before radiographic alterations become evident, making it a crucial diagnostic tool, particularly in patients without established axial involvement [37]. As recommended by the European Alliance of Associations for Rheumatology (EULAR), ultrasound significantly enhances the early identification of peripheral joint and enthesis involvement in SpA, complementing clinical evaluation.

Given the diagnostic advantages of ultrasound in these inflammatory rheumatic diseases, its potential application in non-inflammatory conditions such as FM remains largely unexplored. This study highlights the potential of advanced ultrasonographic techniques, particularly blob analysis, in assessing structural muscle alterations in FM, proposing a novel imaging-based approach for its evaluation. Accordingly, the use of quantitative ultrasonographic techniques has expanded in musculoskeletal imaging, as demonstrated in various studies [41–43]. In one such study, it was highlighted as a valuable, noninvasive tool for diagnosing chronic thyrotoxic myopathy by reliably assessing muscle parameters, such as thickness, cross-sectional area, and echointensity, offering significant insights into muscle quality and pathology [41]. In another study, B + M-mode ultrasound was used to assess swallowing mechanics in stroke patients with dysphagia, identifying key predictors such as tongue and geniohyoid muscle movements and the hyoid-larynx approximation, demonstrating its value in clinical screening [42].

Blob analysis, as a quantitative assessment tool, has been utilized in studies to characterize echointensity and structural alterations in musculoskeletal tissues [44]. Kumbhare et al. conducted a quantitative ultrasound study of the upper trapezius muscle in 15 healthy individuals and 17 patients with myofascial pain. This study demonstrated that the blob area and blob count were effectively differentiated between the two groups. These differences may indicate structural changes such as taut bands, abnormal fiber alignment, or intramuscular fat deposits. In line with these previous findings, the blob alterations observed in our study support the reliability of blob analysis as a valuable method for assessing differences between healthy and pathological muscle tissues.

One of the most important findings of our study was the identification of pain-VAS as a significant predictor of total blob size/mm² in the regression analysis. This suggests that among the various parameters derived from blob analysis, total blob size/mm² may hold particular clinical relevance. Its strong association with pain severity implies that it could serve not only as a supportive diagnostic indicator but also as a valuable parameter in monitoring disease progression or evaluating treatment response in patients with FM. Consequently, incorporating such quantitative parameters into clinical workflows may enhance the precision and consistency of patient monitoring in FM. Although several correlations between ultrasonographic parameters and clinical measures were statistically significant, the relatively modest R values suggest that the effect sizes are small to moderate. Therefore, these findings should be interpreted cautiously, especially in light of the multifactorial and heterogeneous nature of fibromyalgia.

Few studies have evaluated muscle structure in FM using ultrasonography. Umay et al. assessed muscle thickness and cross-sectional areas in patients with FM and reported significant reductions in these parameters, except for the deltoid muscle, when compared to healthy individuals [45]. Their findings suggested that the muscle atrophy observed in FM cannot be solely attributed to physical inactivity, as structural muscle changes are evident even in the early stages of the disease. Research involving both newly diagnosed and established patients with FM indicates that these muscle alterations are more likely driven by disease-specific mechanisms than by inactivity alone. Furthermore, the lack of a significant correlation between physical mobility and muscle measurements supports the hypothesis that structural changes in the FM muscles are primarily associated with the pathophysiological processes of the disease itself, rather than being secondary to reduced physical activity [46]. In line with these findings, Mirza et al. recently demonstrated that FM patients showed significantly reduced thickness of lumbar multifidus muscles. These structural changes were closely associated with impaired muscle endurance, increased fatigue, and decreased functional mobility, further supporting the role of musculoskeletal involvement in FM [47].

Therefore, studies focusing on muscle assessment in FM highlight the significant role of evaluating muscle structures in understanding the pathophysiology, diagnosis, and follow-up stages of the disease. Future research on FM, incorporating ultrasonography with larger sample sizes, diverse muscle group analyses, histopathological validations, and advanced imaging techniques, has the potential to enhance the objective and reliable assessment of tender points, offering clinicians a more effective diagnostic tool than traditional methods such as palpation and patient-reported scales.

Quantitative ultrasonography has emerged as an advanced and promising technique for detailed muscle analysis that offers valuable insights into disease-specific structural changes. Considering the frequent involvement of the trapezius muscle in FM and its association with tender points, this study focused on evaluating this muscle group. The major strength of our study is its contribution to the development of an objective and clinically applicable biomarker, which could enhance diagnostic accuracy, facilitate disease monitoring, and guide more targeted therapeutic interventions in FM.

There are several limitations to this study that should be considered when interpreting the results. Due to the cross-sectional nature of the study, it is not possible to determine whether the observed muscle alterations are a consequence of FM or if they might precede its development. Clarifying this relationship requires future longitudinal research to explore changes over time. The sample included only female participants, which limits the applicability of our findings to the broader FM population, including male patients. Although the condition is more commonly diagnosed in women, it also occurs in men, and their exclusion prevents a more comprehensive understanding of possible sex-based differences in muscle characteristics.

This research was confined to the assessment of the upper trapezius muscle. Since FM is a condition characterized by widespread musculoskeletal pain, evaluating additional muscle groups could offer more holistic insight into the extent and nature of muscle involvement. While we identified significant associations between ultrasound-based muscle changes and clinical measures such as pain and central sensitization, it is important to note that these findings do not establish a direct or disease-specific relationship. FM is widely believed to involve dysfunction of the nervous system, rather than being a primary muscle disorder. Mechanisms involving altered neural processing, autonomic regulation, and small fiber neuropathy are thought to play critical roles in the symptomatology of the disease, and may secondarily affect muscle tissue.

Although the potential influence of commonly prescribed medications such as pregabalin, duloxetine, and amitriptyline was acknowledged, we did not adjust for their effects in the statistical analysis. These agents could impact muscle physiology or alter pain perception, and future studies should aim to control for medication use in order to isolate disease-related changes more precisely.

Finally, quantitative ultrasonography analyses have the potential to provide measurable assessments for FM, thereby supporting its diagnosis, follow-up, and treatment. Central sensitization plays a significant role in the clinical symptoms and muscle structural changes within the complex mechanisms of FM.

## Data Availability

The data associated with this study are not publicly available but can be obtained from the corresponding author upon reasonable request.

## Declarations

### Congress abstract publication

A preliminary version of this study was presented at the 30th National Congress of Physical Medicine and Rehabilitation (Türkiye Fiziksel Tıp ve Rehabilitasyon Derneği) as an oral presentation: Meral HB, Rezvani A, Tolu S, Üşen A. “Fibromiyalji Sendromunda Trapez Kasının Kantitatif Ultrason Analiz Sonuçlarının Hastalık Parametreleri İle İlişkisinin Değerlendirilmesi”. *30. Ulusal Fiziksel Tıp ve Rehabilitasyon Kongresi*, 2024. Abstract No: S-038. The abstract is available at: https://www.tftr.org.tr/dosya/8d83702d-689d-4a4f-8fe3-47df7c83aea1.pdf.

### Disclaimer

No part of this manuscript, including text and graphics, has been copied or published elsewhere in whole or in part. No AI tools were used for writing or editing this manuscript.
